# Commentary: Involvement of LDL and ox-LDL in Cancer Development and Its Therapeutical Potential

**DOI:** 10.3389/fonc.2022.891352

**Published:** 2022-05-02

**Authors:** Uffe Ravnskov, Kilmer S. McCully

**Affiliations:** ^1^ Independent Researcher, Lund, Sweden; ^2^ Pathology and Laboratory Medicine Service, Veterans Affairs Boston Healthcare System, Boston, MA, United States

**Keywords:** cancer, LDL-cholesterol, ox-LDL, lipid-lowering treatment, microorganisms, immune system

## Introduction

As demonstrated by Deng et al. (1), the association between cancer and LDL-cholesterol is an utterly complicated issue. Many studies have shown that high LDL-C is associated with cancer, whereas other studies have found the opposite. In a previous review (2) we have shown that one of the causes of the conflicting findings is that few have realized that the lipoproteins participate in the immune system by adhering to and inactivating almost all types of microorganisms and their toxic products (3) and that 15–20% of human cancers may have a viral or bacterial etiology (4). However, as metastasing cancer cells need much cholesterol, its lowering may perhaps be beneficial in patients with advanced cancer.

## Low Cholesterol May Cause Cancer

Several cohort studies have found that cancer patients have low cholesterol. A common explanation is that cancer cells need cholesterol. However, in nine studies the authors had followed more than 140,000 individuals for 10 - >30 years. After having excluded patients who suffered from cancer during the first four years, they found that cancer appearing during the observation period was inversely associated with cholesterol measured at the start (2). Furthermore, at least five cholesterol-lowering trials have resulted in cancer (5–9). In 4S and HPS (5, 6), the differences between the treatment and the control groups were insignificant, but they become significant if the two trials are calculated together (2). At least four case-control studies have shown that cancer patients have been treated more often with statins than controls. In one of them, almost twice as many patients with lymphoid malignancies had been treated with statins (10). In the other three, which included 2650 patients with prostate cancer, the cancer patients had been treated significantly more often with statins (11–13). Furthermore, in a study of 83 patients with bladder cancer, the tumor became more aggressive in 53% of those who took statins but only in 18% of the non-users (14). These findings contradict the idea that the association between statin treatment and low cholesterol is due to liver damage.

Animal experiments are in accord as well. In a review of data from preclinical studies with lipid-lowering drugs, Newman and Hulley concluded that these drugs produced cancer in rodents after a short time at serum levels close to those achieved in clinical trials (15).

When microorganisms are covered with LDL, they are taken up by macrophages (2), and as macrophages kill the microorganisms by oxidation, it is obvious that LDL may become oxidized as well. The association between cancer and ox-LDL may therefore be caused by infections with carcinogenic microorganisms.

## Discussion

Many follow-up studies and trials have shown that statin-users suffer less often from cancer than non-users. However, several studies have shown that 40-90 per cent of statin-treated patients stop taking their medication after a year or two (16). As most patients who are prescribed cholesterol-lowering treatment have high LDL-C whereas most of the non-treated controls have normal or low LDL-C, it is impossible to know whether the better outcome in these follow-up studies was due to statin treatment or to the patient’s high LDL-C, because none of these studies have asked the patients whether they have continued their statin treatment.

Another bias was presented by Agnoli et al. (17). They followed a large number of hospital patients in Italy between 1993 and 2008 after having measured their lipid values and found that LDL-C at the start was associated with the number who suffered from colon cancer during the observation period. As statin treatment was introduced in the early nineties, many among those with the highest values must have been prescribed such treatment. It is therefore impossible to know whether their cancer was caused by their abnormal lipid values or by statin treatment A more accurate appraisal is therefore to relate the number of cancer cases to the achieved blood cholesterol concentration, as in the study by Matsuzaki et al. (18). They followed 47 294 hypercholesterolemic patients on a low dose simvastatin. Six years later, the number of cancer deaths was more than three times higher among those whose total cholesterol was <160 mg/dl compared with those whose cholesterol was normal or high. A better method is also to measure the lipids at the time of diagnosis as performed by Garrido et al. They measured the lipids of 237 patients remitted for prostate biopsy. About half of them had prostate cancer and their LDL-C was significantly lower than that of those without cancer. However, the finding was not adjusted for other risk factors

According to a meta-analysis of 26 statin trials by the Cholesterol Treatment Trialists’ Collaboration (19), cancer was not seen significantly more often in the treatment groups. However, almost all statin trials have lasted only five years or less, and except for breast cancer, non-melanoma skin cancer and prostate cancer, it takes a much longer time before exposure to carcinogenic chemicals results in easily diagnosed cancers. For instance, it may take several decades before smoking results in bronchial cancer.

Furthermore, most trials did not record non-melanoma skin cancer, the cancer type that is most easy to diagnose. However, in three of the trials mentioned above where statin treatment resulted in cancer, the type of cancer was mentioned. In two of them it was non-melanoma skin cancer (5, 6) and in one of them it was breast cancer (7). In accordance, the number of non-melanoma skin cancer in USA increased dramatically after the introduction of the statins (20).

A possibility is that cholesterol-lowering may protect against metastases. In the CARE trial (7), significantly more women in the treatment group suffered from breast cancer, but in a recent study, 360 women with breast cancer were followed for 23 years (21). After exclusion of those with prediagnostic statin use and those with breast cancer in situ, those who had been prescribed statin treatment during the observation period suffered less often from breast cancer recurrence and lived longer than the untreated. Unfortunately, the authors did not examine whether those on statin treatment had continued the treatment.

A theoretical explanation of the pro-carcinogenic effect of low blood cholesterol is the mitochondrial dysfunction and toxicity caused by loss of thioretinaco ozonide, the active site of oxidative phosphorylation, from the mitochondrial permeability transition pore, the opening of which is inhibited by cholesterol (22, 23).

We think that the benefit of cholesterol-lowering treatment is questionable because this treatment may cause many other serious side effects (24), and most of 38 follow-up studies of patients and healthy people have shown that those with high LDL-C live just as long or longer than people with normal or low LDL-C; none of them found the opposite (25, 26)). It is possible that statin treatment may inhibit the growth of metastases, but this issue requires further investigation.

## Author Contributions

All authors listed have made a substantial, direct, and intellectual contribution to the work and approved it for publication.

## Conflict of Interest

The authors declare that the research was conducted in the absence of any commercial or financial relationships that could be construed as a potential conflict of interest.

## Publisher’s Note

All claims expressed in this article are solely those of the authors and do not necessarily represent those of their affiliated organizations, or those of the publisher, the editors and the reviewers. Any product that may be evaluated in this article, or claim that may be made by its manufacturer, is not guaranteed or endorsed by the publisher.
